# A stonemason with accelerated silicosis in the setting of tumour necrosis factor alpha inhibitor therapy

**DOI:** 10.1002/rcr2.171

**Published:** 2016-07-04

**Authors:** Timothy Baird, Michael Putt, Andrew Dettrick

**Affiliations:** ^1^ Department of Respiratory and Sleep MedicineNambour General HospitalNambourAustralia; ^2^ Department of Respiratory and Sleep MedicinePrincess Alexandra HospitalBrisbaneAustralia; ^3^ Department of PathologyNambour General HospitalNambourAustralia

**Keywords:** Silicosis, stonemason, TNF‐alpha inhibitor

## Abstract

We present the case of a 26‐year‐old stonemason with accelerated silicosis in the setting of treatment for psoriasis with the tumour necrosis factor alpha (TNF‐alpha) inhibitor adalimumab. Accelerated silicosis is an important occupational lung disease with a poor prognosis and limited treatment options [1]. Although the exact pathogenesis remains unknown, it is suggested that secretion of cytokines, including TNF‐alpha, plays a central role in disease progression [1,2]. Importantly, however, TNF‐alpha inhibitors, in addition to resulting in an increased risk of infection, are also now being seen to cause interstitial lung disease [3,4]. To our knowledge, this is the first documented patient to develop silicosis whilst on TNF‐alpha inhibitor therapy. This case challenges the theory behind TNF‐alpha's exact role in the pathogenesis of silicosis and lung fibrosis, highlights the importance of monitoring individuals with both occupational and drug exposures, and illustrates the increasing difficulties physicians face in investigating patients with pulmonary infiltrates and multiple possible aetiologies.

## Introduction

Silicosis is one of the more common occupational lung diseases seen worldwide. Accelerated silicosis, however, is a more aggressive and rare form of the disease that occurs within 10 years of exposure and often leads to progressive massive fibrosis (PMF) with significant morbidity and mortality 1. Although the exact pathogenesis remains unclear, secretion of cytokines such as TNF‐alpha is thought to play an important role in inflammation and the development of fibrosis 2. As a result, it has been theorized that TNF‐alpha inhibitors may provide a treatment option for various causes of pulmonary fibrosis, including silicosis 5. Interestingly, however, TNF‐inhibitors are being increasingly used in a variety of rheumatological and other conditions with the adverse pulmonary effects of interstitial lung disease in addition to mycobacterial and other infections 3, 4. To date, however, there are no reported cases of silicosis occurring in the setting of concurrent TNF‐alpha inhibitor therapy.

## Case Report

A 26‐year‐old male stonemason was referred with a 6‐month history of unproductive cough, exertional dyspnoea, 3 kg weight loss, and infiltrates on plain chest X‐ray. He had a background history of skin psoriasis that was well controlled on 12 months of adalimumab, a TNF‐alpha inhibitor. He was a lifelong non‐smoker and had been working as a stonemason for the past five years. He reported daily exposure to silica dust and only intermittently wore a protective mask. Although he had travelled through tuberculosis endemic regions, he denied any known contact exposure.

**Figure 1:**
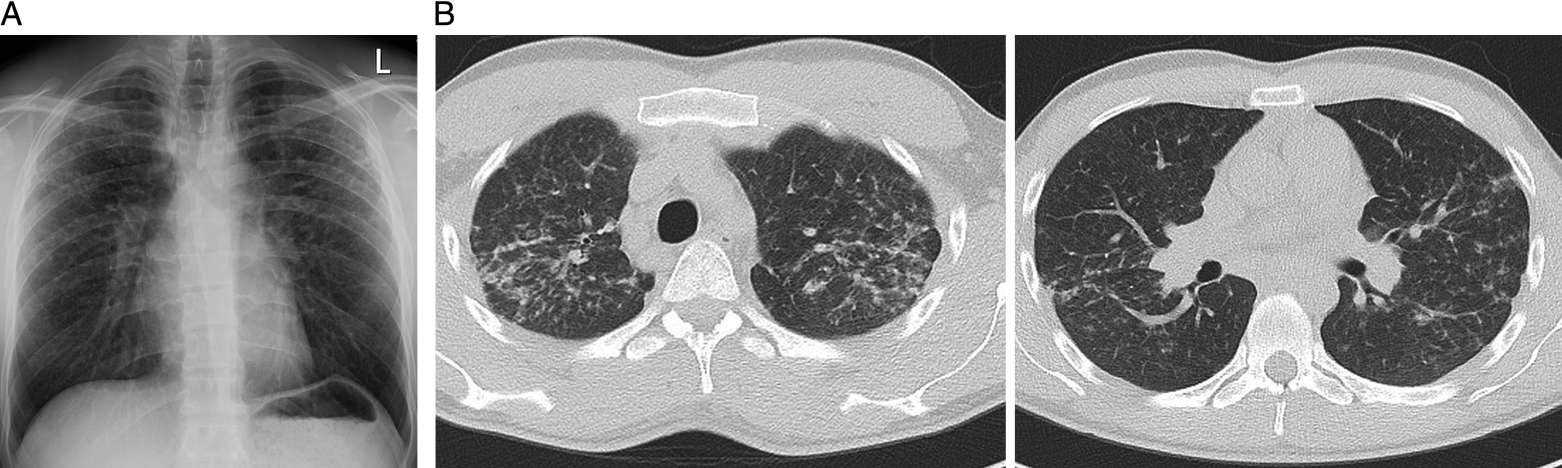
(A) Chest X‐ray showing bilateral upper lobe predominant infiltrates and (B) high‐resolution computed tomography scans demonstrating bilateral upper lobe reticulonodular infiltrates with fibrosis.

Examination was unremarkable with normal oxygen saturations, a clear chest to auscultation, no clubbing or lymphadenopathy, no evidence of pulmonary hypertension, and no signs of connective tissue disease apart from his skin psoriasis. Initial chest X‐ray on specialist presentation demonstrated bilateral upper lobe predominant infiltrates with loss of volume, suggestive of fibrosis (Fig. 1A). His lung function tests showed a moderate restrictive ventilatory deficit with a mild reduction in gas transfer. He underwent a high‐resolution computed tomography scan (HRCT) that confirmed bilateral upper lobe predominant reticulonodular changes with fibrosis as well as prominent mediastinal and hilar lymphadenopathy (Fig. 1B).

At this point, the differential diagnosis consisted of possible silicosis, sarcoidosis, drug‐related interstitial lung disease, connective tissue‐related interstitial lung disease, and infection—specifically reactivation of pulmonary tuberculosis. He underwent a bronchoscopy with bronchioalveolar lavage (BAL), a transbronchial lung biopsy (TBLBx), and an endobronchial ultrasound transbronchial needle aspiration (EBUS‐TBNA). He also underwent a full connective tissue disease screen and was informed to avoid further silica exposure and to cease his adalimumab.

The bronchoscopy and CTD‐screen were non‐diagnostic but importantly returned a negative result for acid‐fast‐bacilli and culture, did not show any evidence of granulomatous inflammation consistent with sarcoidosis or granulomatous infection, and did not demonstrate any specific interstitial lung disease pattern.

**Figure 2:**
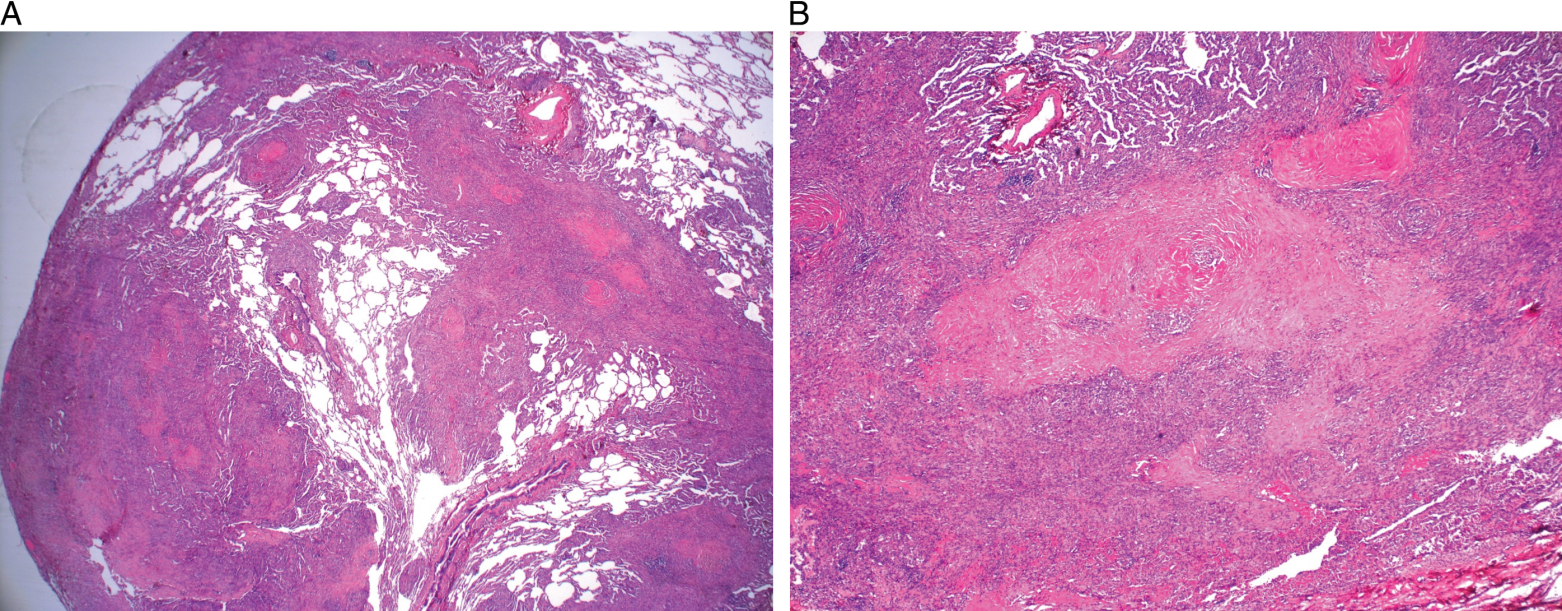
(A) Histology of lung wedge biopsy showing extensive fibrosis and inflammation with nodular pattern along lymphatic routes (haematoxylin and eosin stain [H&E], original magnification ×20) and (B) histology of lung wedge biopsy showing nodules of typical whorled fibrosis centrally with a cellular periphery (which contains macrophages, lymphocytes, and silica particles) (H&E, original magnification ×100).

He then underwent a surgical lung biopsy, which demonstrated silicosis with fibrosis (Fig. 2A,B). His case was discussed at a designated Interstitial Lung Disease Multi‐Disciplinary Meeting, and given the combination of his history, radiology, lung function, and histology, he was diagnosed as having accelerated silicosis with significant fibrosis.

The patient was told to avoid any further silica exposure and was commenced on prednisolone 0.5 mg/kg daily with a plan to slowly wean. Unfortunately, despite complete avoidance of any further silica and the introduction of the steroid sparing agent methotrexate, his radiology and lung function continued to decline. He is now likely developing PMF and is awaiting review for consideration of possible lung transplantation in the future.

## Discussion

Although reported cases of silicosis are declining worldwide, the morbidity and mortality remains high, with limited treatment options. The usual course involves many years of exposure that leads to "chronic silicosis"; however, a rare and aggressive form of "accelerated silicosis" can be seen in patients with less then 10 years of exposure 1. The pathogenesis of this disease remains poorly understood but is thought to revolve around the immunological response of the host with increased secretion of various cytokines, including TNF‐alpha 2. The disease often leads to PMF and has an extremely poor prognosis with no known treatment options other than consideration of transplantation 1.

Moreover, we are seeing an increasing number of biological agents, including the TNF‐alpha inhibitors being developed for the treatment of rheumatological, malignant, and other inflammatory conditions. In addition to these agents putting patients at an increased risk of mycobacterial and other infections, they are continually being shown to cause various patterns of interstitial lung disease 3, 4. Interestingly however, due to their suppressive mechanism of action, they have been proposed and trialled as possible treatment options for various inflammatory and fibrotic lung conditions, although this remains controversial 5.

To the knowledge of the authors, there are no reports in the literature of any patients who have developed chronic or accelerated silicosis in the setting of therapy with a TNF‐alpha inhibitor. This case challenges the theory of increased TNF‐alpha secretion as a potential driver behind disease progression and fibrosis in silicosis and outlines the likely more complex immunological interactions that are at play in the pathogenesis of fibrotic lung diseases.

Finally, in this particular patient, the numerous possible diagnoses due to his multiple exposures made confirming the eventual diagnosis a challenge. This dilemma is becoming increasingly seen by respiratory physicians. Close monitoring and ongoing research of these new biological agents, in addition to maintaining appropriate prevention and monitoring of individuals at risk of occupational lung disease, is paramount.

## Disclosure Statements

No conflict of interest declared.

Appropriate written informed consent was obtained for publication of this case report and accompanying images.
